# Hepatitis C Virus Infection in the Elderly in the Era of Direct-Acting Antivirals: Evidence from Clinical Trials and Real Life

**DOI:** 10.3390/tropicalmed8110502

**Published:** 2023-11-18

**Authors:** Nicola Pugliese, Davide Polverini, Ivan Arcari, Stella De Nicola, Francesca Colapietro, Chiara Masetti, Monica Ormas, Roberto Ceriani, Ana Lleo, Alessio Aghemo

**Affiliations:** 1 Department of Biomedical Sciences, Humanitas University, 20072 Pieve Emanuele, MI, Italy; nicola.pugliese@humanitas.it (N.P.); davide.polverini@humanitas.it (D.P.); ivan.arcari@humanitas.it (I.A.); francesca.colapietro@humanitas.it (F.C.); ana.lleo@humanitas.it (A.L.); 2 Division of Internal Medicine and Hepatology, Department of Gastroenterology, IRCCS Humanitas Research Hospital, 20089 Rozzano, MI, Italy; stella.denicola@humanitas.it (S.D.N.); chiara.masetti@humanitas.it (C.M.); monica.ormas@humanitas.it (M.O.); roberto.ceriani@humanitas.it (R.C.)

**Keywords:** HCV, antiviral therapy, elderly, drug–drug interactions, special populations

## Abstract

The introduction of direct-acting antiviral agents (DAAs) into clinical practice has revolutionized the therapeutic approach to patients with chronic hepatitis C virus (HCV) infection. According to the most recent guidelines, the first line of treatment for HCV infection involves the use of one of three pan-genotypic DAA combinations, sofosbuvir/velpatasvir (SOF/VEL), glecaprevir/pibrentasvir (GLE/PIB), and sofosbuvir/velpatasvir/voxilaprevir (SOF/VEL/VOX). These drugs have been shown to be effective and safe in numerous clinical trials and real-world studies, but special populations have been neglected. Among the special populations to be treated are elderly patients, whose numbers are increasing in clinical practice. The management of these patients can be challenging, in particular due to multiple comorbidities, polypharmacotherapy, and potential drug–drug interactions. This narrative review aims to summarize the current scientific evidence on the efficacy and safety of DAAs in the elderly population, both in clinical trials and in real-life settings. Although there is still a paucity of real-world data and no clinical trials have yet been conducted in the population aged ≥ 75 years old, some considerations about the efficacy and safety of DAAs in the elderly can be made based on the results of these studies. The pan-genotypic associations of DAAs appear to be as efficacious and safe in the elderly population as in the general population; this is both in terms of similar sustained virologic response (SVR) rates and similar frequencies of adverse events (AEs). However, further studies specifically involving this patient population would be necessary to confirm this evidence.

## 1. Introduction

The global prevalence of hepatitis C virus (HCV) infection is estimated to be about 0.7% of the world’s population, corresponding to 56.8 million infections, with new serosurveillance and treatment helping to reduce the burden of the disease [1]. However, HCV infection remains a leading cause of cirrhosis, hepatocellular carcinoma (HCC), liver transplantation, and liver-related mortality, with many people unaware of their status [2]. Another important feature of HCV infection is extra-hepatic manifestations, such as non-Hodgkin’s lymphoma, cardiovascular disease, mixed cryoglobulinemia, and neurological and psychiatric disorders [3]. HCV infection disproportionately affects different generations, with an important peak among people born between 1945 and 1965 (the “baby boomer” generation), who are now entering their seventh and eighth decades of life [4]. Older patients are more likely to be infected with genotype 1b than younger patients, where the predominant genotypes are 1a and 3. Interestingly, in the “baby boomer” generation, older cohort members (1946–1955) have a higher probability of HCV genotype 1b infection, while younger ones have a higher likelihood of genotypes 1a and 3 [5]. In terms of liver damage, HCV infection is commonly associated with a regular progression of fibrosis. Factors associated with accelerated fibrosis include alcohol consumption, male sex, and increasing age. No strong association with viral genotypes has been described [6,7,8]. Before the era of direct-acting antivirals (DAAs), the treatment of HCV infection was based on the use of pegylated interferon (PegIFN) and ribavirin (RBV). PegIFN is associated with many adverse effects, which are even more relevant and severe in elderly patients. In addition, the increased number of comorbidities in these patients often prevented them from receiving this therapy [9]. Consequently, age is an important contraindication to IFN-based regimens, because it is also associated with higher rates of drug discontinuation and reduced sustained virological response (SVR) rates [10]. The approval of DAAs has radically changed the management of HCV, making it possible to treat the vast majority of patients and achieve SVR rates of over 90%, even in difficult-to-treat populations [11]. Today, the most commonly used DAAs in clinical practice are the first-line combinations sofosbuvir/velpatasvir (SOF/VEL) and glecaprevir/pibrentasvir (GLE/PIB), and the second-line combination sofosbuvir/velpatasvir/voxilaprevir (SOF/VEL/VOX), all of which are pan-genotypic [12]. The first combination consists of a non-structural protein 5B (NS5B) inhibitor (sofosbuvir) and an NS5A inhibitor (velpatasvir); the second consists of an NS3/4A protease inhibitor (glecaprevir) and an NS5A inhibitor (pibrentasvir); the last combines sofosbuvir and velpatasvir with the protease inhibitor voxilaprevir [13,14,15]. DAAs are associated with several potential drug–drug interactions (DDIs), which may result in either decreased DAA concentrations and a consequent loss of efficacy, or increased levels of both DAAs and co-medications, which may lead to drug toxicity. Older patients have higher rates of polypharmacy, making the management of HCV infection in this subgroup of patients more challenging. It is therefore imperative to conduct a thorough risk assessment for DDIs prior to initiating therapy for HCV infection [16]. Statins and antipsychotics are the most commonly prescribed co-medications that present DDIs with DAAs, and this is often managed through dose adjustments or temporary discontinuation. Interestingly, the overall higher pill burden does not appear to affect the achievement of SVR [17,18]. Moreover, considering the World Health Organization’s current goal of HCV elimination by 2030, towards which only a few countries are actually on course, the treatment of C virus infection in elderly patients is a key issue [1]. In this review, we discuss the use of DAAs in elderly patients, focusing on the currently available data from both clinical trials and real-world studies, evaluating their efficacy in achieving SVR, their safety profile, and the benefits of treating this specific population.

## 2. Methods

A literature search was conducted on PubMed for articles published in the last two decades, from 1 January 2003 to 1 June 2023, using the keywords HCV, hepatitis C virus, antiviral therapy, direct-acting antivirals, elderly, geriatric, sofosbuvir/velpatasvir (SOF/VEL), glecaprevir/pibrentasvir (GLE/PIB), and sofosbuvir/velpatasvir/voxilaprevir (SOF/VEL/VOX).

## 3. Efficacy and Safety

There are no clinical trials specifically evaluating the use of DAAs in the elderly population, and most data are based on a real-world studies with small and heterogeneous cohorts (Table 1). A study evaluating the safety and efficacy of DAAs in 170 HCV-infected patients aged 80 years or older (mean age 82.3 years, range 80–90), reported an SVR rate of 98.8% (168/170), with no difference in efficacy between the different DAA regimens used (sofosbuvir-based regimens used in 55.9% of patients, GLE/PIB in 25.3%, elbasvir/grazoprevir (EBR/GZR) in 14.1%, ombitasvir/paritaprevir/ritonavir (OMB/PAR/RTV) ± dasabuvir (DAS) in 4.7%). DAA-based regimens were also shown to be safe, with 45 patients (26.5%) reporting mild adverse events (AEs) and only two patients reporting serious non-treatment-related AEs [19]. Similar data were reported in another study evaluating the efficacy and safety of DAA-based regimens in 138 patients aged 70 years or older (mean age 77 years, range 70–95). Sofosbuvir-based regimens were used in 49.4% of patients, EBR/GZR in 25.3%, GLE/PIB in 24.6%, and OMB/PAR/RTV + DAS in 0.7%. Although more common than in the previous study, reported by 37% of patients, the AEs were mostly mild, the most common being nausea (11%) and asthenia (16%), with only two patients experiencing severe AEs due to DDIs. An overall SVR of 94.2% (130/138) and 98.4% (130/132) was observed in the ITT and PP analyses, respectively. Five patients did not achieve SVR: one patient discontinued treatment due to a serious AE (a 76-year-old cirrhotic man with diverticular bleeding), three patients died during follow-up, and only one patient had a virological relapse, a 74-year-old cirrhotic man. A subsequent sub-analysis of the compared patients aged ≥ 80 years and patients aged < 80 years found no statistically significant difference in either their baseline characteristics or treatment outcome, with no differences in the incidence of AEs (*p* = 0.60) or treatment efficacy (*p* = 0.20) [20]. With the advent of pan-genotypic DAAs, international guidelines have recommended their use for the treatment of all HCV-infected patients [12,21]. SOF/VEL, GLE/PIB, and SOF/VEL/VOX are the main drug combinations currently used worldwide because they have few DDIs, do not require treatment monitoring, and are effective in inducing SVR across all HCV genotypes. Numerous clinical trials have demonstrated their efficacy in the vast majority of patients, and these data have been confirmed in real-world observational studies. However, there is currently a paucity of data on the use of these regimens in specific subpopulations, including elderly patients.

## 4. Clinical Trials

To our knowledge, no clinical trials have focused on patients aged ≥ 75 years, and the elderly population was usually under-represented in most trials, although old age was never an exclusion criterion.

### 4.1. Sofosbuvir/Velpatasvir

The ASTRAL-1, ASTRAL-2, and ASTRAL-3 pivotal trials demonstrated an excellent clinical response to SOF/VEL treatment, with an overall SVR ≥ 95% in the ITT analysis (99% (618/624), 99% (133/134), and 95% (264/277) in ASTRAL-1, ASTRAL-2, and ASTRAL-3, respectively). Although patients aged ≥ 75 were under-represented, it is important to note that no patient older than 70 years failed to achieve SVR. The safety data were also reassuring, as the most common adverse events were mild (fatigue, headache, nasopharyngitis, and nausea) and led to the premature discontinuation of treatment in only one patient in ASTRAL-1 (a 52-year-old man who experienced an anxiety attack), and one patient in ASTRAL-2 (a 57-year-old man who experienced an anxiety attack after receiving a dose of the study drug). More importantly, in ASTRAL-1, there was no significant difference found in the AE rates between the SOF/VEL and the placebo groups (78% and 77%, respectively) [22,23], and treatment with SOF/VEL was associated with an improvement in quality of life in all patients, including the elderly [24,25]. These data have been confirmed in subsequent studies. An African single-arm prospective study evaluated the response to 12 weeks of SOF/VEL in a predominately GT4-infected African population. The median age was 64 years (IQR 51–74). SVR was achieved in 59 of 61 patients, with an SVR of 97% (95%, CI 89–99) in the ITT analysis. Only two patients did not achieve SVR: a 46-year-old non-cirrhotic woman infected with GT4r with three NS5A resistance-associated substitutions and one NS5B resistance-associated substitution, and a 51-year-old non-cirrhotic man infected with GT4 with two NS5A resistance-associated substitutions. In the study, 59 (97%) patients reported at least one mild AE, the most common being hypertension (39%), headache (30%), dizziness (20%), abdominal pain (18%), and arthralgia (16%), while 18 (30%) patients reported moderate to severe AEs, although none of the severe AEs were related to the study drug [26]. More clinical trials are available in the literature, but few tend to include older patients. For example, no patients older than 75 years were enrolled in ASTRAL-4 or ASTRAL-5 [27], and in an Indian study evaluating the efficacy of SOF/VEL, only 7 (5%) of 129 patients were aged ≥ 65 years [28].

### 4.2. Sofosbuvir/Velpatasvir/Voxilaprevir

POLARIS-1, POLARIS-2, POLARIS-3, and POLARIS-4, the pivotal studies for SOF/VEL/VOX, all included patients aged ≥ 75 years. In POLARIS-1, SOF/VEL/VOX was administered for 12 weeks and the SVR rate was compared to a matched placebo group consisting of GT1-infected patients previously treated with a regimen containing an NS5A inhibitor. The mean age of the treatment arm was 58 years (range 27–84). Overall, the SVR rate in the treatment arm was 96% (253/263) in the ITT analysis, and of the ten patients who did not achieve SVR, none were ≥ 70 years of age. In POLARIS-4, which compared SOF/VEL/VOX with SOF/VEL in patients with GT1, GT2, and GT3 infection previously treated with a DAA regimen but not an NS5A inhibitor, an overall SVR of 98% (178/182) was observed in the SOF/VEL/VOX arm, with only one virologic failure, a 62-year-old cirrhotic man with GT2 infection. Interestingly, a subgroup analysis showed a slightly higher SVR in patients aged ≥ 65, although the difference was not statistically significant [29]. Similar results were reported in POLARIS-2 and POLARIS-3, with an SVR in the ITT analysis of 95% (477/501) in a population with a mean age of 53 years (range 18–78) and an SVR of 96% (106/110) in a population with a mean age of 54 years (range 25–75), respectively. As in POLARIS-1 and POLARIS-4, the SVR rates in POLARIS-3 were higher in patients aged ≥ 65, though were still not statistically significant [30]. Regarding safety, 78% of patients receiving SOF/VEL/VOX in POLARIS-1 experienced an AE compared to 70% of patients receiving the placebo. The most common AEs in the treatment arm were headache (25%), fatigue (21%), diarrhoea (18%), and nausea (14%), the same as in the placebo group. One patient who received SOF/VEL/VOX in POLARIS-1, a 59-year-old woman, and three patients in the placebo group discontinued treatment prematurely due to AEs. No patient receiving SOF/VEL/VOX in POLARIS-2, POLARIS-3, or POLARIS-4 discontinued treatment due to AEs. Despite the large number of studies evaluating the efficacy and safety of SOF/VEL/VOX in addition to the POLARIS trials, the majority of the study populations did not exceed 75 years of age [31,32,33].

### 4.3. Glecaprevir/Pibrentasvir

In the ENDURANCE-1 study, GT1-infected non-cirrhotic patients were divided into two arms and treated with GLE/PIB for 8 and 12 weeks. The median age of the first arm was 53 years (range 19–84), while the median age of the second arm was 52 years (range 21–77). The SVR rate in the ITT analysis was 99.1% (348/351) in the 8-week arm and 99.7% (351/352) in the 12-week arm, while the SVR rate in the PP analysis was 100% regardless of the treatment duration. The safety profile of GLE/PIB was similar in patients treated for 8 or 12 weeks, with the most common AEs (occurring in at least 10% of patients) being headache, pruritus, and fatigue. Serious AEs were reported in 1% to 2% of patients in each treatment group; none were considered to be related to the study drug [34]. These promising data were further underscored in the CERTAIN-1 study, a multicentre, open-label, phase 3 study evaluating the safety and efficacy of GLE/PIB in GT1-infected Japanese patients with and without cirrhosis. The median age was 64 years (range 21–86) in the non-cirrhotic arm treated with 8 weeks of GLE/PIB, and 73 years (range 48–85) for the cirrhotic arm treated with 12 weeks of GLE/PIB; interestingly, the elderly population was well represented, with patients ≥ 75 years of age accounting for 20% (26/129) and 42% (16/38) of the study population in the non-cirrhotic and cirrhotic arms, respectively. All patients in both arms achieved SVR, with only one patient in the 8-week arm lost to follow-up after achieving SVR at 4 weeks after the end of treatment. In both arms, 60% of patients experienced AEs, with 23% and 18% of AEs in the non-cirrhotic and cirrhotic arms, respectively, being considered drug-related. Despite this, no serious AEs were reported and no patients discontinued treatment prematurely due to AEs [35]. The high SVR rates achieved with GLE/PIB in GT1-infected patients have also been confirmed in other genotypes. For example, in CERTAIN-2, GT2-infected non-cirrhotic Japanese patients achieved SVRs of 97.8% (88/90) and 100% in the ITT and PP analyses, respectively, with one patient lost to follow-up and one patient discontinuing treatment due to nausea and vomiting. The median age of the population was 57 years (range 26–83), with 10 patients (11%) over the age of 75 [36]. In EXPEDITION-1, GT1-, GT2-, GT4-, GT5-, and GT6-infected patients with compensated cirrhosis and a mean age of 60 years (range 26–88) achieved an SVR of 99% (145/146), with only one relapse on post-treatment, a GT1a-infected patient with a history of non-response to pegylated IFN plus ribavirin [37]. These data are reassuring, but although there are numerous clinical trials validating the efficacy and safety of GLE/PIB, older patients remain an under-represented category, as they are usually a clear minority or not included in the study at all [38,39,40,41].

## 5. Real-World Data

Real-world data are critical to understanding treatment efficacy and safety in everyday clinical practice, particularly in patient populations that may be excluded from or under-represented in clinical trials, such as older patients.

### 5.1. Sofosbuvir/Velpatasvir

In the real world, sofosbuvir-based regimens have been shown to be effective and safe in the elderly [19,20]. A Taiwanese observational study evaluated the safety and efficacy of SOF/VEL and GLE/PIB, which were examined in a population of 1356 patients (614 treated with SOF/VEL and 742 with GLE/PIB) with a mean age of 63.31 ± 14.24 years for the SOF/VEL arm and 62.12 ± 12.95 years for the GLE/PIB arm. The SVR rates of the GLE/PIB and SOF/VEL arms were 98.9% (710/718) and 99.5% (581/584), respectively, in the PP analysis, and 95.7% (710/742) and 94.6% (581/614), respectively, in the ITT analysis. All patients older than 70 years achieved SVR (215/215) in the SOF/VEL arm, while the SVR rate in the GLE/PIB arm was 99.1% (210/212). In terms of safety, the vast majority of AEs were mild to moderate, with the most common in the GLE/PIB and SOF/VEL groups being pruritus (17.4% vs. 2.9%), abdominal discomfort (5.8% vs. 4.4%), dizziness (4.2% vs. 2%), fatigue (3.1% vs. 2.9%), and elevation of total bilirubin to 1.5–3 × ULN (5.3% vs. 2.9%) [42]. SOF/VEL was also effective in patients with decompensated cirrhosis (defined as a Child–Pugh score grade B or C): a Japanese study evaluating SVR in patients with decompensated cirrhosis showed an SVR of 92.2% (59/64) with a 95% CI of 82.7–97.4 via PP analysis, and a subgroup analysis showed no significant difference between SVR rates in patients younger than 75 years and patients ≥ 75 years of age (*p* = 0.341), with SVRs of 95.1% (39/41) and 87.0% (20/23), respectively [43], while another study, with a population equally divided between younger and older patients (mean age of 68 years, range 40–87) with decompensated cirrhosis, showed an SVR of 90.2% (74/82) in an ITT analysis [44].

### 5.2. Sofosbuvir/Velpatasvir/Voxilaprevir

Few real-world studies evaluating the response to SOF/VEL/VOX are currently available [45,46], and few data are available in the elderly population. A retrospective, longitudinal, multicentric, real-life study evaluated SVR rates in 179 patients treated with SOF/VEL/VOX for 12 weeks; the median age was 57 years (range 18–88). In the ITT analysis, 162/179 patients (91%) achieved SVR, with no statistically significant difference (*p* = 0.78) between the age of the group that did not achieve SVR (seven patients, median age 55 years (range 50–73)) and the group that did (162 patients, median age 57 years (range 18–88)). Although not statistically significant, it is interesting to note that all patients aged ≥ 75 years of age achieved SVR. Overall, SOF/VEL/VOX was also safe, with 28 patients (16%) experiencing drug-related AEs of mild to moderate severity, the most common of which were fatigue (6%), hyperbilirubinemia (6%), and anaemia (3%). Eleven (6%) serious AEs occurred in eight patients, none of which were considered drug-related [13]. Another study evaluated the efficacy of SOF/VEL/VOX in treatment-experienced patients, including 490 with GT1 (219 (44.7%) ≥ 65 years), 20 with GT2 (10 (50%) ≥ 65 years), 51 with GT3 (10 (19.6%) ≥ 65 years), and 12 with GT4 (five (41.7%) ≥ 65 years). Patients aged ≥ 65 years of age achieved an SVR of 90.0% (190/211) and 94.8% (184/194) in the GT1 group, 90.0% (9/10) and 88.9% (8/9) in the GT2 group, 100.0% (7/7) and 100.0% (7/7) in the GT3 group, and 100.0% (5/5) and 100.0% (5/5) in the GT4 group in the ITT and PP analyses, respectively, with no significant difference between the 55–66 years group and the <55 years group [47]. Further studies are needed to confirm the efficacy and safety of SOF/VEL/VOX in the population aged ≥ 75 years.

### 5.3. Glecaprevir/Pibrentasvir

Real-world data confirmed the high SVR rates and overall safety of GLE/PIB observed in clinical trials [48]. The MARS post-marketing observational study evaluated GLE/PIB in a large population of Italian patients (337 at baseline, 321 with SVR available at study end). The SVR rate was 99.4% (319/321), with a 77-year-old cirrhotic man with a relapsing GT3 infection and an 84-year-old non-cirrhotic woman who discontinued therapy early due to nausea being the only patients who did not achieve SVR. Similar results were reported in the elderly subpopulations, with an SVR rate of 98.1% in patients aged ≥ 65 years. In terms of safety, 18/337 patients (5.3%) had AEs that were reasonably related to the study drug, of which two (both < 75 years) had AEs of grade ≥ 3 severity (0.6%) [49]. Only one study identified age at treatment (*p* = 0.031) as a baseline clinical factor discriminating between treatment success and treatment failure; specifically, older patients appear to have a greater chance of benefiting from GLE/PIB treatment [50]. This was not confirmed in subsequent studies, where SVR rates were not statistically correlated with age at treatment [15,51]. Few specifically designed studies have reported the efficacy and safety of GLE/PIB in the elderly population, mainly from Europe and Asia. A multicentric, retrospective study from Japan [52] evaluated the virological response and safety of GLE/PIB in younger (<75 years) and older (≥75 years) patients infected with GT 1 and GT2 (only one patient aged < 75 years had GT 3). Patients with decompensated liver cirrhosis and active hepatocellular carcinoma (HCC) were excluded from the study. The study showed no significant difference in response between the two groups, with SVR rates of 95.8% and 98.6% in the older group and of 92% and 98.9% in the younger group, respectively, based on ITT and PP analyses. Of the three patients who did not achieve SVR, only one was in the older group, a 75-year-old woman with GT2a infection who had a virologic relapse. There were no severe AEs, but four patients discontinued treatment due to AEs; all of them were in the younger group. These data were a further confirmation of the results of two previous Japanese studies [53,54]. In both studies, the response to treatment was evaluated in younger (<75 years) and older (≥75 years) patients and no significant differences were found: in particular, in the first study, the SVR rates were 96.8% (241/249) and 99.6% (237/238) in the younger group and 98.3% (56/59) and 100% (56/56) in the older group in the ITT and PP analyses, respectively. Moreover, even when taking into account parameters such as the presence of compensated cirrhosis (SVR 94% in the younger group vs. 94.4% in the older group in the ITT analysis), different genotypes (SVR 96.6% and 96.9% in the younger group vs. 100% and 96.6% in the older group in the ITT analysis for GT1 and GT2, respectively), prior DAA treatment (SVR 94.4% in the younger group vs. 100% in the older group in the ITT analysis), or severe chronic kidney disease (SVR 100% in the younger group vs. 100% in the older group in the ITT analysis), a difference could not be found. Regarding safety, although the incidence of pruritus was lower in the younger group, the rates of grade ≥ 3 adverse events and other adverse events were not significantly different between the two groups. Outside Asia, a recent multicentric Italian study retrospectively evaluated the efficacy and safety of GLE/PIB in a population aged ≥ 75 years [55]. Treatment with GLE/PIB achieved SVR rates of 97.9% (558/570) and 99.6% (558/560) in the ITT and PP analyses, respectively. In addition, there were no safety concerns, with only 10 (2%) patients discontinuing treatment prematurely and 48 (8%) patients experiencing AEs. In addition, SVR rates were not significantly influenced by gender, treatment duration, prior IFN-based therapy, or the presence of liver cirrhosis. Similar results were reported in another recent study that showed no significant difference between the older (≥65 years) and the younger group treated with GLE/PIB, with SVR rates of 97.3% and 97.9% in the ITT analysis, respectively [56].

**Table 1:** Summary table of the main studies reported in this review. GT, genotype; SVR, sustained virologic response; ITT, intention to treat; PP, per protocol; AE/SAE, adverse events/severe adverse events; CI, confidence interval; SOF/VEL, sofosbuvir/velpatasvir; SOF/VEL/VOX, sofosbuvir/velpatasvir/voxilaprevir; GLE/PIB, glecaprevir/pibrentasvir. * failure was reported only in patients < 75 years old.

Drug	Study	GT	Number of Patients	Mean Age	Number of Patients ≥ 75 Years	SVR by ITT	SVR in Patients ≥ 75 Years	Discontinuation Due to AE/SAE
	**Clinical trials**							
SOF/VEL	ASTRAL-1 [22]	1, 2, 4, 5, 6	624	54 years (range 18–82)	Not available	99% (618/624)	100% *	1 (52 year old)
	ASTRAL-2 [22]	2	134	57 years (range 26–81)	Not available	99% (133/134)	100% *	1 (57 year old)
	ASTRAL-3 [23]	3	277	49 years (range 21–76)	Not available	95% (264/277)	100% *	0
	SHARED-3 [26]	4	61	64 years (range 51–74)	Not available	97% (59/61)	100% *	2 (46 year old and 51 year old)
SOF/VEL/VOX	POLARIS-1 [29]	1, 2, 3, 4, 5, 6	263	58 years (range 27–84)	Not available	96% (253/263)	100% *	1 (59 year old)
	POLARIS-2 [30]	1, 2, 3, 4, 5, 6	501	53 years (range 18–78)	Not available	95% (477/501)	100% *	0
	POLARIS-3 [30]	3	110	54 years (range 25–75)	Not available	96% (106/110)	100% *	0
	POLARIS-4 [29]	1, 2, 3, 4	182	57 years (range 24–85)	Not available	98% (178/182)	100% *	0
GLE/PIB	ENDURANCE-1 [34]	1	351—8-week arm	53 years (range 19–84)	Not available	99.1% (348/351)	Age not available	1 (age not available)—12-week arm
			352—12-week arm	52 years (range 21–77)		99.7% (351/352)		
	CERTAIN-1 [35]	1	129—non-cirrhotic arm treated for 8 weeks	64 years (range 21–86)	20% (26/129	99% (128/129)	100% *	0
			38—cirrhotic arm treated for 12 weeks	73 years (range 48–85)	42% (16/38)	100%	100% *	1 (age not available)
	CERTAIN-2 [36]	2	90—non-cirrhotic arm treated for 8 weeks	57 years (range 26–83)	11% (10/90)	97.8% (88/90)	100 *	1 (age not available)
			18—cirrhotic arm treated for 12 weeks	70 years (range 49–85)	33% (6/18)	100%	100 *	1 (age not available)
	EXPEDITION-1 [37]	1, 2, 4, 5, 6	146	60 years (range 26–88)	Not available	SVR 99% (145/146)	100 *	0
	**Real world data**							
SOF/VEL	Chang et al. [42]	1, 2, 3, 6	614	63.31 ± 14.24 years	35% (215, ≥ 70 years old)	94.6% (581/614)	100%	0
	Tada et al. [43]	1, 2	65	69 years old (range 60–79)	35.9% (23)	92.2% (59/64) (PP analysis)	87.0% (20/23)	0
SOF/VEL/VOX	Degasperi et al. [13]	1, 2, 3, 4	179	57 years (range 18–88)	Not available	91% (162/179)	100% *	0
GLE/PIB	Chang et al. [42]	1, 2, 3, 6	742	62.12 ± 12.95	28.6% (212, ≥70 years old)	95.7% (710/742)	99.1% (210/212)	5 (age not available)
	MARS post-marketing trial [49]	1, 2, 3, 4, 5, 6	334	56 years (range 19–87)	32.3% (108, ≥ 65 years old)	99.4% (319/321) (PP analysis)	98.1% (105/108) (PP analysis)	1 (84 year old)
	Komaki et al. [52]	1, 2, 3	271	65 years (range 26–88)	26.5% (72)	92%	95.8%	4 (all 4 patients < 75 years old)
	Watanabe et al. [53]	1, 2, 3	308	65 years (range 26–96)	19.6% (59)	96.8% (241/249)	98.3% (56/59)	10 (3 patients < 75 years old)
	Pugliese et al. [55]	1, 2, 3, 4, 5, 6	570	80 years (range 75–97)	100% (570)	-	97.9% (558/570)	10

## 6. Prognostic Benefit of SVR

Achieving SVR is the main goal of HCV treatment. In patients with mild to moderate fibrosis, HCV eradication stops the progression of liver damage and prevents the risk of HCV transmission, allowing the patient to be discharged from further follow-up [57]. In patients with advanced liver fibrosis or cirrhosis, the risk of HCC, liver decompensation, and all-cause mortality is lower with HCV eradication therapy compared to with no treatment [57,58]. However, in patients who achieve SVR and have advanced liver disease, the risk of death is independently associated with the severity of the liver disease. This association explains why, in this subgroup of patients, SVR appears to have less impact on liver-related death than in patients with mild or moderate fibrosis [59]. Achieving SVR is associated with significant improvements in liver function tests and transaminases. The improvement in liver function is mainly achieved in the first two years after SVR. Decreases in alpha-fetoprotein levels, as well as increased platelet counts and albumin levels, are also well described after eradication therapy [57,60,61]. Re-evaluation with transient elastography one year after achieving SVR shows a significant reduction in liver stiffness (LS), which is even greater in cirrhotic patients. However, subsequent assessments at longer time intervals do not show further improvements in cirrhotic patients. A possible explanation for this lack of improvement over time may be that the eradication of HCV determines an immediate reduction in liver inflammation [62]. In patients with clinically significant portal hypertension (CSPH), HCV eradication has been shown to contribute to a reduction in the hepatic venous pressure gradient (HVPG), probably due to the architectural remodelling that occurs after virus clearance. This important outcome is observed in most patients with CSPH 48 weeks after achieving SVR, and it is predictive of both a reduced risk of hepatic decompensation and a prolonged survival [63]. HCC is the most common liver-related event following SVR [64]. According to scientific society guidelines, patients with advanced fibrosis or cirrhosis should be surveyed for HCC every six months, because SVR reduces but does not eliminate the risk of HCC [12]. Patients who develop HCC after SVR have better liver function and a lower tumour stage at presentation, which could also be due to a better surveillance. These favourable characteristics determine a better survival in patients with SVR, if compared to viraemic patients. HCV treatment after HCC diagnosis has also been shown to improve survival [65]. Some factors have been associated with progression to HCC after HCV treatment, such as diabetes, metabolic dysfunction-associated steatotic liver disease (MASLD), and alcohol consumption [66]. Regarding liver steatosis, HCV is known to contribute to lipogenesis, lipoprotein synthesis, and fatty acid oxidation, so its eradication can improve steatotic liver disease [67]. Other factors that have been described as associated with HCC development include age, male sex, genotype 1 infection, history of HCC, and pre-treatment alpha-fetoprotein levels [58,68]. The eradication of HCV infection has also been shown to be associated with a significant reduction in the risk of cardiovascular and all-cause mortality [59,61,69]. Moreover, the current literature is also showing an improvement in brain bioelectrical activity and neurocognitive functions after HCV eradication [70,71]. Life years gained is another important outcome that has been evaluated in many studies, and they show a greater benefit in younger patients and those with more advanced liver fibrosis, with a progressive reduction with increasing age. In the elderly, the most significant effect in life-year gain is achieved in patients with significant fibrosis, together with a sensitive improvement in quality of life [72]. Of course, cost-effectiveness analyses have been performed to determine the real impact of HCV treatment in the elderly in order to guide public health decisions. According to these analyses, the decision on whether or not to offer treatment to elderly patients should be based on their frailty, from a holistic point of view, and not just on their age or liver disease burden [73].

## 7. Conclusions

The advent of DAAs has radically changed the management of HCV, making it possible to treat almost all infected patients. Currently, DAAs are considered the standard of care, but data on their efficacy and safety in certain subgroups of the HCV-infected population are still limited. This is of particular concern in the elderly population, which is growing and difficult to manage. In addition, they are often receiving polypharmacotherapy with potential DDIs, and they tend to present with a more severe stage of liver disease. The data currently available, from both clinical trials and real-world studies, are limited, but all agree on the safety and efficacy of DAAs in the elderly population. The achievement of SVR appears to be comparable to that in the general population, and no significant difference in AEs and treatment discontinuation has been demonstrated.
